# Mothers’ experiences of a telephone based breastfeeding support intervention after discharge from neonatal intensive care units: a mixed-method study

**DOI:** 10.1186/s13006-017-0142-9

**Published:** 2017-12-19

**Authors:** Jenny Ericson, Renée Flacking, Camilla Udo

**Affiliations:** 1 Department of Women’s and Children’s Health, Uppsala University, Uppsala, Sweden; 2 Centre for Clinical Research Dalarna, Nissersväg 3, S-79182 Falun, Sweden; 3 Department of Paediatrics, Falu Hospital, Falun, Sweden; 4 School of Education, Health and Social Studies, Dalarna University, Falun, Sweden

**Keywords:** Breastfeeding, Mixed method, Neonatal, Preterm infant, Support, Telephone, Thematic network analysis

## Abstract

**Background:**

After discharge from a neonatal intensive care unit (NICU), many mothers of preterm infants (gestational age < 37 weeks) experience a lack of support for breastfeeding. An intervention study was designed to evaluate the effects of proactive (a daily telephone call initiated by a member of a breastfeeding support team) and/or reactive (mothers could call the breastfeeding support team) telephone based breastfeeding support for mothers after discharge from the NICU. The mothers in the intervention group had access to both proactive and reactive support; the mothers in the control group only had access to reactive support. The aim of this study was to explore the mothers’ experiences of the proactive and reactive telephone support.

**Methods:**

This study was a qualitatively driven, mixed-method evaluation using three data sources: questionnaires with qualitative open-ended questions, visual analogue scales and telephone interviews. In total, 365 mothers contributed data for this study. The qualitative data were analysed with an inductive thematic network analysis, while the quantitative data were analysed with Student’s t-test and the chi-square test.

**Results:**

Proactive support contributed to greater satisfaction and involvement in breastfeeding support. The mothers who received proactive support reported that they felt strengthened, supported and secure, as a result of the continuous care provided by staff who were knowledgeable and experienced (i.e., in breastfeeding and preterm infants), which resulted in the global theme ‘*Empowered by proactive support*’. The mothers who received reactive support experienced contradictory feelings; some felt secure because they had the opportunity to call for support, whereas others found it difficult to decide when and if they should use the service, which resulted in the global theme; ‘*Duality of reactive support*’.

**Conclusion:**

There were positive aspects of both proactive (i.e., greater satisfaction and feelings of empowerment) and reactive support (i.e., the opportunity to call for support); however, the provision of reactive support alone may be inadequate for those with the greatest need for support as they are the least likely to access it.

**Trial registration:**

NCT01806480 on 5 March 2013.

## Background

Breastfeeding a preterm infant (gestational age [GA] < 37 weeks) may be perceived as a positive experience [1] in the sense that it helps the mother feel emotionally close to her infant and strengthens the mother-infant bond [2]. However, breastfeeding may also be a complex and stressful process. Feeding may be challenging for several reasons, including latching difficulties, the need to express breast milk and inadequate support from healthcare services [1, 3]. Preterm infants must often be admitted to a neonatal intensive care unit (NICU) for a few days up to several months. In such cases, breastfeeding develops in an unfamiliar medical context. In NICUs, there is a focus on breast milk intake and breastfeeding because of the beneficial effects of breast milk on growth and development [4]. Compared with infants born at term, preterm infants exhibit immature breastfeeding behaviour depending on their GA at birth; thus, the time until the preterm infant can be exclusively breastfed varies [5]. In NICUs, mothers are offered varying degrees of professional breastfeeding support, which are often determined by the motivation, skills and presence of the nurses [6]. After discharge from NICUs in Sweden, mothers are referred to the public health service or peer support for breastfeeding support. All mothers are offered the opportunity to make regular visits to children’s healthcare centres after discharge from the NICU, where breastfeeding support is part of the service. For additional breastfeeding support, mothers can seek support at other healthcare settings or via peer support. For some mothers, NICU discharge presents a new beginning and an opportunity for breastfeeding, whereas others experience a lack of support for continued breastfeeding [6]. In a Swedish study, the parents of preterm infants reported a lack of follow-up counselling after discharge, especially for milk intake and breastfeeding [7]. Hence, there is a risk that mothers of preterm infants will discontinue breastfeeding during the first months at home with their infants [8].

A systematic review showed that professional breastfeeding support is important for continued breastfeeding [9]. To facilitate breastfeeding, adequate support should be person-centred and should include trusting relationships and continuity of care [10]. Person-centred support, which involves a professional’s sympathetic presence, engagement and shared decision making, has the potential to increase the recipient’s satisfaction with, and involvement in the support process and to promote health and wellbeing [11].

A randomised controlled trial (RCT) was conducted to evaluate the effects of proactive breastfeeding support (in which the mothers received a daily telephone call) compared with only reactive support (in which the mothers had to initiate the telephone call themselves) for mothers of preterm infants after discharge from a NICU [12]. The mothers allocated to the proactive support group could also obtain reactive support if they wished. The mothers in the proactive group showed statistically significantly lower parental stress compared with the mothers in the reactive group, although no statistically significant effects for breastfeeding were found [13]. Qualitative aspects are important in intervention evaluations because they have the potential to contribute to a deeper understanding of how the users experienced the intervention and to identify how an intervention may be improved [14]. The aim of this study was therefore to describe mothers’ experiences of proactive and reactive breastfeeding telephone support after discharge from a NICU.

## Methods

### Design

This study was an evaluation of an RCT using a qualitative-driven, concurrent, embedded mixed-method approach in which data from questionnaires and telephone interviews were used [15, 16]. An embedded approach can be defined as one in which data collection and analysis are linked and may be used to examine subjects’ experiences of trial interventions [15].

### Participants and setting

The RCT was conducted from March 2013 to December 2016 and involved 493 breastfeeding mothers of preterm infants (born <37 weeks GA) who had spent >48 h in a NICU and were discharged from one of six NICUs in Sweden. The mothers were randomised to either proactive support (intervention group) or reactive support (control group). The proactive support group received daily telephone calls from a member of a breastfeeding support team (BST) from day 1 to day 14 after discharge from the NICU. In addition, the mothers in this group had the option to call someone in the BST during the same period (i.e., to receive reactive telephone support). In the reactive support, the mother had to initiate telephone contact with the BST from day 1 to 14 after discharge from the NICU if they had questions or wanted to share something [12]. The study aimed to provide continuity of care by providing support from the NICUs during the two first weeks after discharge. Ideally, only a few members of the BST would call each mother, but sometimes this was not possible because of staff work schedules. Of the 231 mothers randomised to proactive support, four mothers also accessed reactive support. In the reactive group, 41 mothers called the BST at some point.

**Fig. 1:**
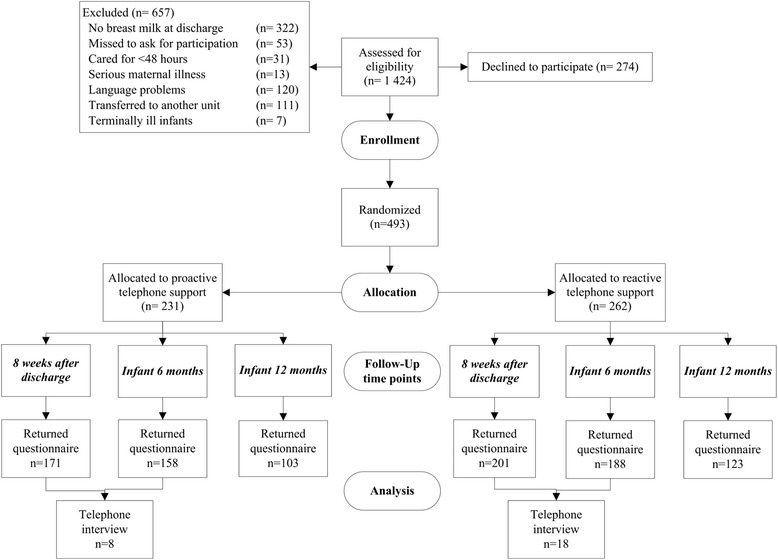
Data collection and the number of participants

All the participating mothers had the opportunity to answer open-ended questions and complete visual analogue scales (VAS) via questionnaires distributed 8 weeks after discharge from the NICU and when the infants were 6 and 12 months postnatal age. At all follow-ups, a letter was attached to the questionnaires distributed to both the proactive (intervention) and reactive (control) groups asking the mothers to participate in an interview regarding their experiences of the telephone support they received (Fig. 1).

During February and March 2016, mothers whose infants had been home for more than two months but less than six months and who had consented to participate in an interview received phone calls from CU (who performed the telephone interviews) and were again invited to participate in a telephone interview. All 26 mothers who were invited (eight in the proactive and 18 in the reactive group) consented to be interviewed. At the time when the mothers were asked to participate in the telephone interview, the researchers were blind to group allocation (i.e., proactive or reactive group).

### Data collection

#### Questionnaire

In the 8-week follow-up questionnaire, the mothers were asked open-ended questions about breastfeeding and breastfeeding support (i.e., proactive and reactive support). The mothers’ satisfaction with their group allocation, their involvement and their satisfaction with the breastfeeding support provided was evaluated using VAS scales. The questions asked were *“How satisfied/involved were you with the support you received via telephone?”* The answers were scored on a 10-cm VAS scale that ranged from very dissatisfied/not at all involved (0) to very satisfied/very involved (10). The questionnaires completed when the infants were six and 12 months postnatal age included open-ended questions about the mothers’ experiences of the telephone support and about infant feeding and breastfeeding support in general such as *“How did you experience the breastfeeding support?”* The written answers to the open-ended questions were pasted into a Word document.

#### Telephone interviews

The interviews were guided by a semi-structured interview guide [17] containing questions such as, *“Could you please tell me how you have experienced the telephone support?” “How has the breastfeeding support matched your personal values regarding breastfeeding?”* At the end of the interviews, the mothers were given an opportunity to express their views, describe experiences or comment on anything they wished. The telephone interviews lasted 7–42 min and were audio-recorded and transcribed verbatim (see Fig. 1 for information regarding data collection and the number of participants).

### Data analysis

#### Statistical analysis of the quantitative questionnaire responses

To study the participants’ satisfaction with group allocation, a chi-square test was used. To analyse the VAS scale scores regarding the mothers’ involvement in and satisfaction with the breastfeeding support and to compare the proactive and reactive groups, Student’s t-tests were calculated. The statistical significance level was set at *p* < 0.05 for all analyses, and calculations were performed with IBM SPSS Statistics for Windows, Version 21.0 (Armonk, NY: IBM Corp).

#### Thematic network analysis of the qualitative data from the interviews and questionnaires

Thematic network analysis, inspired by Attride-Stirling [18], was used to reduce and organise the data systematically and to describe the findings. Thematic analysis is well suited for summarising a text and illustrating the findings [18]. Transcribed text from the interviews and the written responses to the open-ended questions in the questionnaires were combined and read as a whole regardless of group allocation and time-point. The two authors (JE and CU) read the transcribed text several times. As a first step in reducing the text, segments of the text were identified and coded according to the aim of the study. These coded text segments were organised into preliminary basic themes by the first author (JE). The basic themes were discussed among all authors and redefined before they were merged and arranged into five preliminary organising themes. Adhering to the original text expressions, the five organising themes were rearranged and translated from Swedish to English during discussions among all authors before two global themes were deduced. In these later steps in the analysis, the themes were named in English. All the authors critically reflected on all steps in the analysis process, and on all analytical decisions until consensus was reached. JE and RF translated the selected quotes from the telephone interviews and written comments from Swedish to English. In the next step, a professional English translator checked the translated quotes. In the findings, the quotes from the telephone interviews were labelled ‘interview xx’ and the quotes from the written comments were labelled with the mothers’ randomisation code, e.g., ‘F5’.

In this study, the authors JE and RF are paediatric nurses with many years of experience working in NICUs; CU is a social worker by profession with many years of experience in healthcare but not within neonatal care. The authors’ different professional perspectives contributed to both an inside and outside view of breastfeeding support. JE and RF were responsible for the design of the intervention.

## Results

The characteristics of the participating mothers are described in Table 1.

**Table 1:** Characteristics of the participating mothers and their preterm infants

	*Total* (*n* = 493)	*Written comments* *(n = 274)*	*Telephone interview* *(n = 26)*
Participant characteristics	*n* (%)	*n (%)*	*n (%)*
Randomised to			
Proactive support	231 (47)	132 (48)	8 (31)
Reactive support	262 (53)	142 (52)	18 (69)
Maternal age, year; mean (SD)	30 (5.2)	30.5 (5.2)	30 (4.7)
Maternal educational level			
Higher education	258 (52)	160 (58)	15 (58)
Upper secondary school or less	235 (48)	114 (42)	11 (42)
Primipara mothers	278 (56)	164 (60)	16 (61)
Mothers not born in Sweden	46 (9.3)	18 (6.6)	2 (7.7)
Vaginal birth	277 (56)	158 (60)	13 (50)
Infant gestational age at birth, weeks; median (IQR)	34 (2)	34 (3)	34 (1)
Infant gestational age at discharge, weeks; median (IQR)	38 (2)	38 (2)	39 (2)
Length of stay for the infant, days; median (IQR)	23 (21)	23 (20)	25 (22)
Multiple birth	52 (11)	30 (11)	6 (23)
Exclusive breastfeeding			
At discharge	406 (82)	231 (84)	21 (81)
8 weeks after discharge	282 (57)	171 (62)	13 (50)
6 months postnatal age	103 (21)	72 (26)	7 (27)
Partial breastfeeding at 12 months postnatal age*	49 (22)	32 (20)	4 (27)

SD = standard deviation, IQR = interquartile range

*281 mothers agreed to answer the additional questionnaire administered when their infants were 12 months postnatal age

### Satisfaction with group allocation and involvement in and satisfaction with the breastfeeding support intervention according to the quantitative questionnaire responses

Statistically significant differences were found between the proactive and reactive support groups. The mothers in the proactive group were significantly more satisfied with the group allocation than the mothers in the reactive group (98%, *n* = 167 vs. 88%, *n* = 171, *p* < 0.001). Of the 303 mothers who answered the questions on involvement and satisfaction with the breastfeeding support intervention, those in the proactive group felt more involved in the telephone support, mean ± (SD) 8.6 (2.1) vs. 7.2 (2.7), *p* < 0.001, and were more satisfied with the telephone support, 8.8 (1.8) vs. 7.5 (2.5), *p* < 0.001, than those in the reactive group.

### Mother’s narratives regarding proactive and reactive support based on the qualitative data from the telephone interviews and written comments

**Fig. 2:**
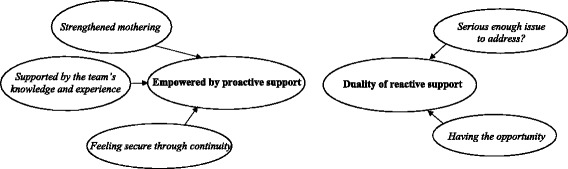
Global and organising themes

In the analysis of the narratives regarding proactive and reactive support, two global themes emerged; *Empowered by proactive support* and *Duality of reactive support*. These two global themes consisted of five organising themes (Fig. 2). Although all the text was read as a whole without any separation between the groups, interviews or written comments, the differences between the proactive and reactive group became evident during the analysis.

### Empowered by proactive support

The mothers who received proactive support reported being strengthened as mothers and supported by the BST’s knowledge and experiences and feeling secure as a result of the continuity of care. The mothers’ descriptions showed that their individual specific needs in breastfeeding and caring for their preterm infants were acknowledged and met by the BST.

#### Strengthened mothering

The mothers were strengthened by the encouragement and confirmation provided by the BST, which the mothers described as an important aspect of the support. The feeling of being a good mother emerged when the mothers received feedback and confirmation of their thoughts and actions.

*“It was a boost for my self-confidence that they really show interest, ask [questions], and care. That is how it felt. Therefore, it was only positive. You may not be able to breastfeed fully, and I’ve been given the support that it’s not unusual*. *.*. *that there are many who may not be able to breastfeed fully. You need to hear that you’re still a good mom with a little baby. Therefore, it was nice to get that support and advice.” (Interview 10).*

The mothers also noted that when the BST paid attention to their specific situation and needs, it contributed to an open discussion in which they perceived the support as meeting their individual needs. This kind of attention helped the mothers feel involved in the support process and made them feel more confident as mothers. The mothers also noted that the BST genuinely seemed to care for them as a person because they took their problems seriously, were concerned about them and treated them with respect.

*“If I had questions, they could answer them and they were very attuned. I thought the whole conversation was modelled after me*. *.*. *like the questions I had and what problems I had and so on. Then there was the encouragement. Sometimes you might not be so eager to continue breastfeeding after so many months of tube feeding and pumping to just get that encouragement, a little pat on the shoulder.” (Interview 20).*

However, when the mothers did not receive individually adapted support and/or felt disregarded, they lacked trust in the BST. As an example of this, one mother described a situation in which she was not listened to:

*“I didn’t feel that great, and I didn’t have much milk, and I was wondering if I could give her formula. She was crying a lot, and I didn’t have enough breast milk. The answer I got was to hand express, and right then, it wasn’t possible. I continued to breastfeed, but she didn’t gain weight. What was important to me was that my baby wouldn’t be sad; if I'm breastfeeding or not doesn’t matter. I would have wanted another answer.” (Interview 15).*

#### Supported by the team’s knowledge and experience

The mothers valued the knowledge and experience of the BST staff members on issues related to breastfeeding and preterm infants. In addition, the mothers were pleased that the staff could answer their questions regarding common problems (e.g., infant digestion and sleeping problems and mastitis) that occurred when the mother returned home. The BSTs were described as knowledgeable and skilled regarding breastfeeding and preterm infants’ health and development, which fostered trust in information and advice that they provided. The mothers compared the support they received from the BST with that of the nurses at the child healthcare centres, whom they felt did not have enough knowledge about preterm infants. The mothers also felt that they had sufficient support from the BST and therefore did not need to seek support elsewhere.

*“I have a preterm baby, and she was tube fed in the beginning, and then I started to breastfeed. I really wanted to continue breastfeeding, and she [the nurse in the BST] helped me, so it went well. You must push yourself in the beginning. She listened and answered my questions. She came with advice and ideas. She was very knowledgeable and could answer everything that I asked and wondered about. She seemed to care and she really shared and wanted it to go well.” (Interview 21).*

Some of the mothers commented that they generally preferred face-to-face support because some breastfeeding problems were too difficult to explain by phone. The mothers expressed an occasional need for more practical help, e.g., having a BST team member come to their home to show them physically how to do or looking at something (e.g., the infant’s latch or a sore nipple) rather having to describe it verbally over the telephone.

*“It would have been good if someone in the breastfeeding support team would have come home to me and helped me when I was breastfeeding.” (T35, 12-month follow-up).*

#### Feeling secure through continuity

The mothers in the proactive group were grateful that someone from the BST called on a regular basis. However, they indicated that 2-weeks’ support was too little and that problems had arisen after the intervention ended. The mothers felt it was a relief to be contacted so they did not have to decide for themselves whether or when to call for support. They felt calm knowing they would receive a telephone call and have the opportunity to ask questions, and they felt secure and supported knowing that someone from the BST would call.

*“Safe start to be called every day.” (F23, 12-month follow-up).*

*“I thought it felt good. In any case it felt good to know that someone was going to call if I had any questions.” (Interview 3).*

The mothers were also grateful that they knew who to turn to if there was a problem and that breastfeeding support was available. If a problem occurred, the mothers felt they could always address it during the next telephone contact with the BST. The mothers mentioned that they discussed minor problems when the BST called; these were problems that they would not otherwise discuss with anyone else. The continuous daily support from the BST helped the mothers feel safe and supported.

*“You felt safe being called because you could always ask. It was something just after I was discharged, I think. It was during the first call that they asked me how it was going, how do you feel, do you feel safe when you're at home and do you have any questions when you are at home? How is the breastfeeding coming along? How is the child doing? Perhaps I needed to hear all that.” (Interview 17).*

However, although most of the mothers considered the continuity of care a positive factor, those who had had negative experiences of staff support during the NICU stay considered the continuity of care a prolonged negative experience. These mothers felt that they were pressured and under stress to breastfeed, and they perceived a general lack of involvement in the provided support.

*“Felt stressed and pressured to breastfeed in the neonatal unit and even after by being called every day.” (Ö14, 8-week follow-up).*

### Duality of reactive support

The narratives revealed that the mothers in the reactive group experienced ambivalence because of the duality of the reactive support. On the one hand, they were grateful to have a telephone number to call if they needed support; on the other hand, they had difficulties deciding whether their problem was serious enough to call the BST.

#### Having the opportunity

The mothers in the reactive group mentioned that they were grateful and felt safe because they had the opportunity to call the BST if they needed help and support. Some of the mothers called the BST when questions or problems occurred (e.g., Is my baby receiving enough breast milk? How should I handle mastitis?). The mothers who called the BST, were satisfied with the support they received and expressed how important it was that the staff listened to their problems. The mothers also felt that the telephone based support was tailored to their unique needs.

*“It suited me well to call. I called when I had problems. It was very nice to have that number to call. So it was great to have a breastfeeding supporter. [I] had mastitis, [so] I had to get help somewhere.”* (*Interview 21).*

However, most of the mothers in the reactive group rarely used the telephone support service. The most common reasons for not using the telephone support service were that breastfeeding was going smoothly, that they did not experience any serious problems, or that they felt very confident in solving their potential problems. Furthermore, some of the mothers who did not use the reactive support stated that the support they received from friends and family, child healthcare centres and the social media was good and that they did not need additional support.

*“A friend is a ‘help mother’ in a peer-support group in Sweden called Help in breastfeeding (In Swedish: Amningshjälpen). If there are any problems, I can contact her.” (SU43, 8-week follow-up).*

#### Serious enough issue to address?

The mothers who were randomised to the reactive support group reported hesitation in calling the BST, largely because they did not know whether their problems were severe or important enough to address. The mothers reported that although they sometimes felt the need for support, they thought that their issue/question was too banal or insignificant to call the BST therefore, they decided to cope on their own. The mothers were also uncertain about when to call the BST and found it difficult to prioritise asking their questions above the other chores/activities that needed to be done. Other reasons for not calling were losing the BST telephone number or even forgetting that the BST existed.

*“You think about things, things that you don’t think are important enough to call and ask. It might be easier if someone calls and just talks a little*. *.*. *then you would ask those questions.” (Interview 18).*

The mothers in the reactive group conveyed that if the BST had used the proactive approach, they would have gained more knowledge, and issues that the mothers had not even acknowledged would have been discussed and possibly addressed.

*“If I had ended up in the other group [the proactive group], maybe I would have learnt more. If anyone had called me every day asking how it was going, then perhaps I would have said, yes, it feels slightly like this or it's slightly like that.” (Interview 18).*

## Discussion

This study describes the experiences of a proactive and reactive telephone based breastfeeding support intervention for mothers of preterm infants after discharge from a NICU. Our results indicate that the mothers who received proactive support were more satisfied and felt more involved in the support than the mothers who received reactive support. The mothers who received proactive support felt empowered as mothers. They felt strengthened as mothers and supported by the BSTs. Moreover, the mothers in the proactive group favoured having someone from the BST calling them at home; rather than having to call someone from the BST. Receiving continuous breastfeeding support contributed to a feeling of security and comfort. The mothers in the reactive support group experienced conflicting reactions: on one hand, having the opportunity to contact the BST contributed to a sense of security. On the other hand, some of the mothers did not use the support even when they felt that they had a legitimate reason to call.

The results show that the mothers who received proactive support felt empowered, which has the potential to increase their resources and reduce stress [19]. Our results confirm the findings of previous studies of mothers of term infants by showing that providing engaging, responsive breastfeeding support helps women feel valued and acknowledged as good mothers [10, 20]. McCormack and McChance’s definition of person-centred care suggests that the outcomes of effective person-centred care are increased satisfaction with and involvement in care and a feeling of wellbeing [11]. In addition, it established a therapeutic relationship between the person and the professional supporter. This definition is in line with the present findings that the mothers in the proactive group were involved in and satisfied with the telephone based support. These mothers also described the importance of the continuity and accessibility of the services provided, which are important components of person-centred support [10, 11]. The present study aimed to provide continuity of care in that the support from the NICU continued after discharge. It was not intended for the continuity of care to be provided by one or two nurses for each mother; however, it is desirable to reduce the number of team members involved with each family. Although the quantitative data set does not reflect it, the mothers in the reactive group experienced breastfeeding support as person-centred, i.e., they felt the support team was sensitive to their needs and treated them as an individual. This observation shows that when the mothers in the reactive group contacted the BST, they felt that support was provided, even though they rarely used the support. The mothers in the reactive group felt significantly less involved in the support process, which may indicate that the proactive approach per se promotes involvement and cooperation. Involvement is an important part of person-centred support; when there is a lack of involvement, there is a risk of a poor relationship between the mother and supporter, which may lead to decisions being made without the mothers’ participation [11]. The value of person-centred support has also been highlighted in a meta-synthesis by Schmied et al. [10], who showed that breastfeeding mothers feel supported when they are listened to with empathy, provided with detailed and realistic information based on their needs and given encouragement and affirmation. Similar to Schmied et al. [10], we found that when support is not adapted to the individual there is a risk that the mothers, regardless of group, will feel pressure and stress instead of guidance and support in breastfeeding.

The differences between the two groups in involvement and satisfaction, together with the empowerment of the mothers and the security of continuity of care experienced by the mothers who received proactive support may partly explain our RCT finding [12] that mothers who received proactive support experienced significantly less parental stress.

Although our study demonstrated that both proactive and reactive support have the potential to contribute to feelings of security and comfort, the meaning of security differed between the groups. For the mothers who received proactive support, security was linked to continuity and knowing that someone was going to call them daily. Such support gave the mothers the opportunity to ask questions, even if they were minor or insignificant. In the reactive group, security was linked to having a telephone number for the BST that they could use if they experienced breastfeeding problems. Our findings show that the mothers used the reactive support to a low extent. The positive aspect of this finding is that many of the mothers did not need additional support; their self-efficacy and/or the support they received was ‘good enough’. However, the cases in which the mothers did not call the BST although they wanted to cause concerns as mothers who need support do not receive it when only reactive support is provided. This raises the question of which mothers contact the BST and ask for support and which do not and, subsequently, who receives support. It may be that mothers with fewer resources do not use the support. A previous study of a telephone hotline that new mothers could call for support showed that mothers used the telephone support and recognised the worth of the service; however, only small numbers of mothers in ethnic minority groups called [21]. Furthermore, in a pilot study evaluating proactive and reactive breastfeeding telephone support that targeted disadvantaged areas, only one woman called in the reactive group [22]. The results of the present study indicate that for mothers in the reactive group, there may be a gap between having the opportunity to call and actually calling.

The strengths of this study are the large number of participating mothers and the mixed-method approach, which included quantitative and qualitative data that complemented each other and drew attention to different perspectives. In this study, qualitative data were used to assess the validity of the quantitative findings and to generate insights [23]. The qualitative and quantitative data were concurrently collected and analysed. Another strength is that this study described experiences from those included in the intervention group as well as those in the control group, i.e., mothers who only received reactive support.

Although the written comments were derived from both groups to the same extent, a weakness is that due to randomisation, the telephone interviews were mainly conducted with mothers who only had experienced reactive support. However, the data collected from the interviews with the mothers in the proactive group were analysed together with written comments, and therefore, the difference in the number of mothers interviewed from each group may not have affected the result.

A limitation of this study is that the qualitative data originated from written open-ended questions and that the interviews were conducted over the telephone instead of face-to-face. This approach may have limited the depth and nuances of the qualitative material. However, conducting telephone interviews and collecting written qualitative material enabled collection of data from a larger sample of mothers than would have been feasible with face-to-face interviews.

## Conclusion

In this study, a telephone based breastfeeding support intervention delivered by a BST was evaluated using a mixed-method design. Differences between the groups were found, showing that proactive support contributed to the mothers feeling more satisfied with the support and being more involved in it. In addition, the mothers felt empowered through strengthened mothering, the teams’ knowledge and experiences and the continuity of care provided via the proactive support. The reactive support was associated with contradictory experiences; the mothers felt safe because they had the opportunity to call for support but also found it difficult to determine when to use the support. Our results indicate that although there were positive aspects of both proactive and reactive support, providing only reactive support may be inadequate for those most in need of support as they may be the least likely to access it.

## Abbreviations

BST: Breastfeeding support team
NICU: Neonatal intensive care unit
RCT: Randomised controlled trial
